# Growth of lithium hydride thin films from solutions: Towards solution atomic layer deposition of lithiated films

**DOI:** 10.3762/bjnano.10.142

**Published:** 2019-07-18

**Authors:** Ivan Kundrata, Karol Fröhlich, Lubomír Vančo, Matej Mičušík, Julien Bachmann

**Affiliations:** 1 Institute of Electrical Engineering, SAS, Dúbravská cesta 9, 841 04 Bratislava, Slovakia; 2 Friedrich-Alexander University of Erlangen-Nürnberg, Dept. Chemie and Pharmacy, Chair ”Chemistry of Thin Film Materials”, Cauerstr. 3, 91058 Erlangen, Germany; 3 Centre of Excellence for Advanced Materials Application SAS, Dúbravská cesta 5807/9, 841 04, Bratislava, Slovakia; 4 STU Centre for Nanodiagnostics, Slovak University of Technology in Bratislava, Vazovova 5, 812 43 Bratislava, Slovakia; 5 Polymer Institute, Slovak Academy of Sciences, Dúbravská cesta 9, 845 41 Bratislava, Slovakia; 6 Saint Petersburg State University, Institute of Chemistry, Universitetskii pr. 26, 198504 St. Petersburg, Russia

**Keywords:** lithiated thin films, lithium hydride, solution atomic layer deposition (sALD)

## Abstract

Lithiated thin films are necessary for the fabrication of novel solid-state batteries, including the electrodes and solid electrolytes. Physical vapour deposition and chemical vapour deposition can be used to deposit lithiated films. However, the issue of conformality on non-planar substrates with large surface area makes them impractical for nanobatteries the capacity of which scales with surface area. Atomic layer deposition (ALD) avoids these issues and is able to deposit conformal films on 3D substrates. However, ALD is limited in the range of chemical reactions, due to the required volatility of the precursors. Moreover, relatively high temperatures are necessary (above 100 °C), which can be detrimental to electrode layers and substrates, for example to silicon into which the lithium can easily diffuse. In addition, several highly reactive precursors, such as Grignard reagents or *n*-butyllithium (BuLi) are only usable in solution. In theory, it is possible to use BuLi and water in solution to produce thin films of LiH. This theoretical reaction is self-saturating and, therefore, follows the principles of solution atomic layer deposition (sALD). Therefore, in this work the sALD technique and principles have been employed to experimentally prove the possibility of LiH deposition. The formation of homogeneous air-sensitive thin films, characterized by using ellipsometry, grazing incidence X-ray diffraction (GIXRD), in situ quartz crystal microbalance, and scanning electron microscopy, was observed. Lithium hydride diffraction peaks have been observed in as-deposited films by GIXRD. X-ray photoelectron spectroscopy and Auger spectroscopy analysis show the chemical identity of the decomposing air-sensitive films. Despite the air sensitivity of BuLi and LiH, making many standard measurements difficult, this work establishes the use of sALD to deposit LiH, a material inaccessible to conventional ALD, from precursors and at temperatures not suitable for conventional ALD.

## Introduction

While the development of electric motors and semiconductor devices is progressing, the pressure on battery development is increasing correspondingly. Rechargeable, and if possible recyclable, batteries are versatile power sources for virtually all mobile devices. The advent of pocket hand-held devices places even stricter demands on the safety of rechargeable batteries. Although increased safety can be achieved using sophisticated and reliable charge-controller circuits, inherent safety is still desirable. Since the hazardous components in lithium-ion batteries are organic solvents used as electrolyte, their exclusion would greatly improve the inherent safety of lithium-ion batteries. Solid-state batteries that are already in use, such as the LIPON battery in which the solid electrolyte consists of nitrogen-doped lithium phosphate, present several shortcomings. One of them is the use of sputtering [1] for the deposition of the thin layers. Inherently, sputtering does not yield coatings with high conformity on non-planar substrates. Low conformity leads to low surface area and thick films are needed to avoid pinholes. This, in turn, leads to low capacity mainly due to the low surface area. The whole concept of a solid-state battery needs to be reconsidered, particularly if we wish to surpass the capacity of current liquid-electrolyte batteries. However, the natural obstacle of upscaling from the nanoscale to macroscopic batteries and large macroscopic capacities cannot be avoided. While a niche use can be found for wholly nanoscale batteries, such as a nanoscale batteries for nanoscale transistors, the scaling issue needs to be addressed for more general applicability. To meet this challenge, the use of atomic layer deposition (ALD) has been proposed [2–3]. The inherent conformity of ALD indeed allows for thinner, conformal, pin-hole free films [4–5].

ALD has been instrumental in enabling the development of nanobatteries, especially when combined with substrates of high surface area, which allow for increased capacity values. One such example is the V_2_O_5_–SnO_2_ nanobattery [6] grown on anodized alumina. ALD can also deposit lithiated films, using precursors such as Li(thd), lithium *tert*-butoxide, and lithium hexamethyldisilazane [7]. Lithium hexamethyldisilazane enabled the direct deposition of deposit Li_2_SiO_3_ using ozone as a secondary precursor [7], at temperatures beginning at 150 °C, which are among the lowest for lithium ALD. Especially interesting is the ALD deposition of the aforementioned LIPON, which is currently the most popular solid-state electrolyte. Two approaches have been demonstrated in 2015. One is a quaternary process [8] adopting the lithium *tert*-butoxide and water process used to deposit Li_2_O. To the cycle additional pulses of trimethylphosphate and nitrogen plasma were added, incorporating phosphorus and nitrogen into the Li_2_O film at 250 °C. In the resulting LIPON films the nitrogen concentration could be varied between 0 and 16.3% [8]. Another approach to deposit LIPON using ALD is to incorporate nitrogen into the phosphorus precursors. Diethyl phosphoramidate has been successfully used in combination with lithium hexamethyldisilazane to deposit LIPON films [9]. The key insight was the use of a nitrogen-containing phosphorus precursor to directly create the P–N bonds. The resulting films grown by this technique at 270–330 °C were amorphous and the nitrogen concentration increased with the process temperature [9]. Despite these progresses, ALD has not yet been adopted to deposit lithium-containing films outside of laboratories, mainly due to the sensitivity of electrochemically active films to water, oxygen, and carbon dioxide. [7].

The deposition of lithiated compounds using conventional ALD uses expensive and complicated precursors, as well as relatively high temperatures. Especially high temperatures can be detrimental for the stability of lithiated films [10], because a part of the Li ions can diffuse [11] into substrates and devices. The use of several highly reactive precursors, such as Grignard reagents, which only exist in solution, could in theory allow for lower temperatures to be used. However, ALD cannot easily work with precursors that only exist in solution, or decompose below 100 °C. A novel ALD technique, namely solution atomic layer deposition [12] (sALD), opens up new ways to overcome these difficulties. In contrast to regular ALD, sALD uses solvents as precursor-carrying media, thereby eliminating the need for complicated gas supply lines and vacuum chambers, vastly simplifying the necessary setup for deposition. Furthermore, there is no need for complex filters for hazardous byproducts, since the liquid waste from the deposition process can be easily caught, neutralized, and the solvent can be recovered by distillation.

sALD opens up the possibility to use *n*-butyllithium, which does not exist in the gas phase, as a precursor for lithiated films [13]. Furthermore, such organolithium precursors in solution are inexpensive and easier to handle, more so than the required volatility of precursors for standard ALD would allow [7]. Because precursors that cannot be used in ALD are used in sALD, reactions that are impossible in the gas phase can be explored, even producing ionic compounds. Here we focus on the reaction of BuLi with water in diethyl ether, exploring the deposition characteristics when using the sALD technique of sequential delivery of precursors in solution.

## Experimental

### Deposition setup

**Figure 1:**
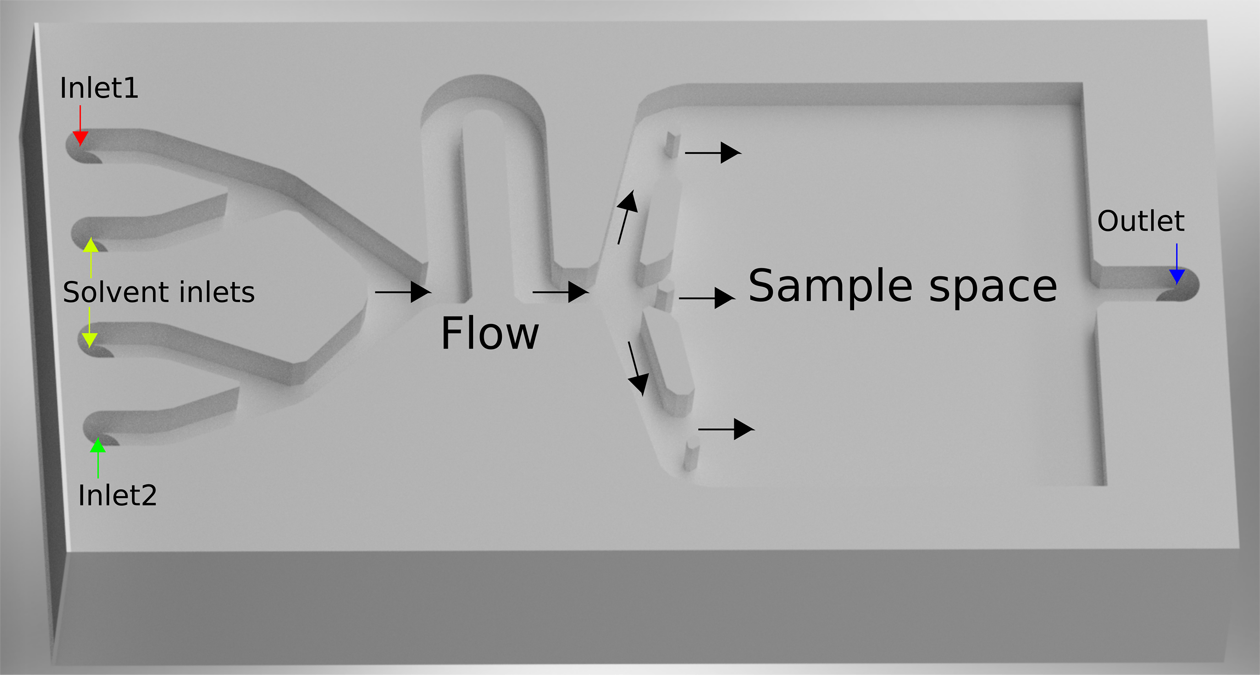
Illustration of the experimental deposition chamber. Top view, without cover. The chamber is closed from the top and then sequentially flooded with solutions.

**Figure 2:**
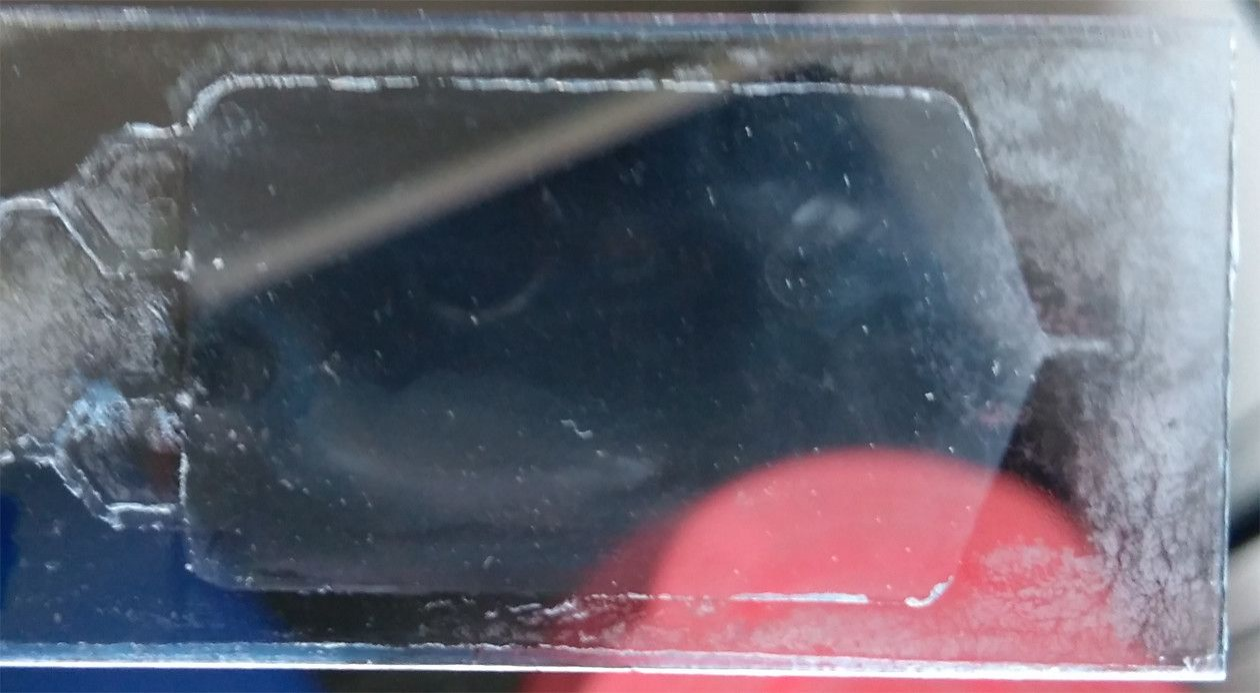
Chamber cover after deposition. The shape of the chamber is outlined by the PTFE paste used for sealing. A white film can be seen on the glass slide.

**Figure 3:**
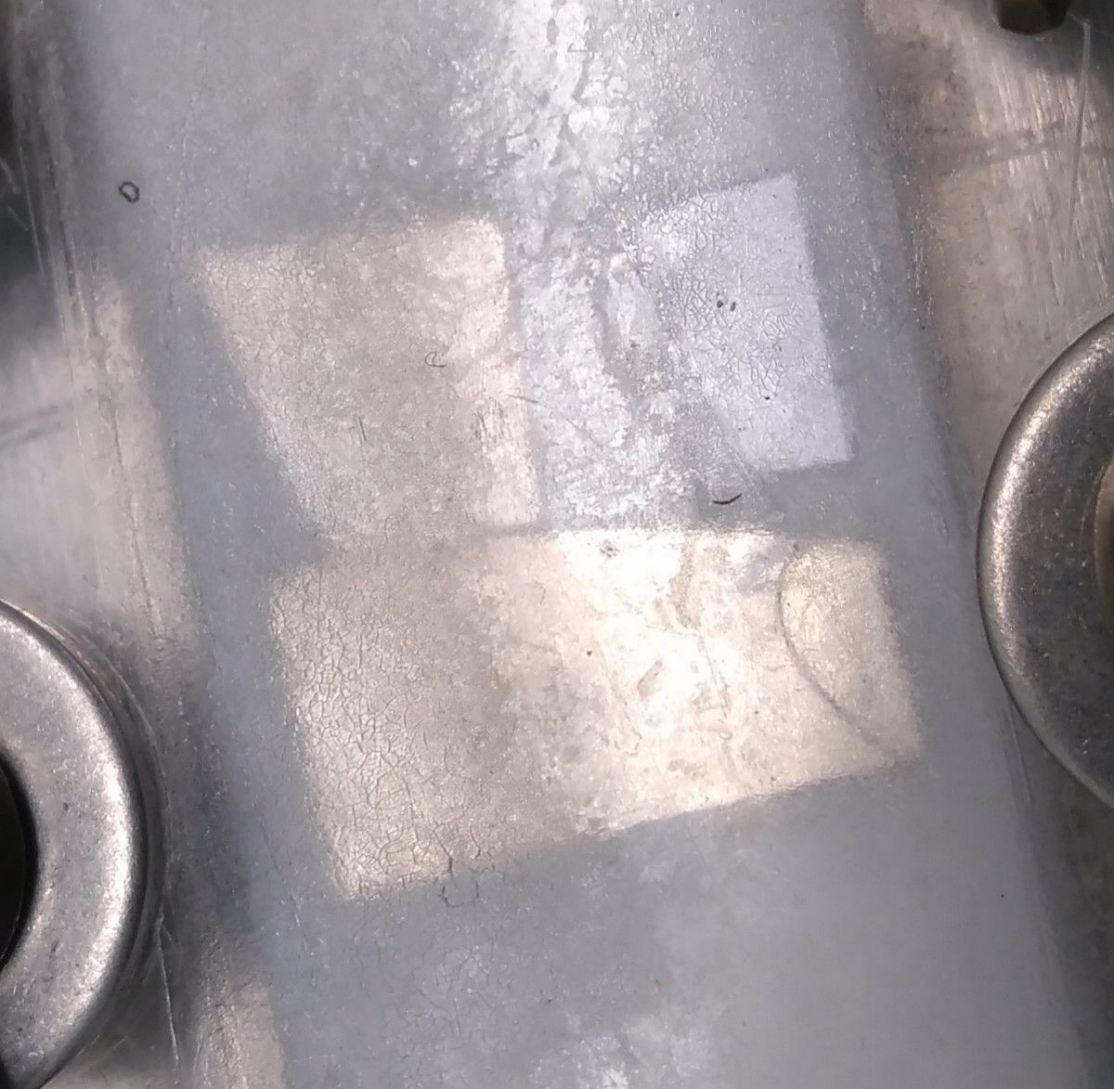
Samples in chamber during deposition, immediately before being taken out of the chamber. The glass slide was held in place by an acryl block, immediately upon removal of the block the chamber unsealed and the films started reacting with air.

Initially, a deposition chamber made out of PTFE, which is shown schematically in Figure 1, was loaded with sample substrates. To close the chamber, a glass slide (see below in Figure 2) was sealed to the chamber with PTFE grease. The solutions were introduced into the chamber with stainless steel threaded pipes, which were connected to threads in the PTFE body of the chamber. Teflon tubes running through peristaltic pumps served as connections between the chamber and the needles in the precursors flasks, which were kept under nitrogen overpressure in a Schlenk line. All depositions were carried out at room temperature. Samples in the chamber during deposition can be seen below in Figure 3.

### Materials and methods

Precursors for the deposition were prepared in an argon-filled moisture-free glovebox, and then handled within a nitrogen-filled Schlenk line. Dry ether (ROTIPURAN 99.5%, p.a., lump), used as the primary solvent, was further dried with pure sodium and molecular sieves. *n*-Butyllithium (2.5 M in hexanes, Sigma) was diluted to 10 mM with diethyl ether. Deionized water was used as the complementary precursor, dissolved in diethyl ether (20 mM). The purging was carried out with nitrogen from the Schlenk line. In order to monitor the surface chemistry, a quartz crystal microbalance (QCM) system was installed behind the exit of the deposition setup with tubing of minimal length (2 cm), effectively allowing the deposition to occur on the QCM crystal as well. The waste pumped out of the QCM chamber was immediately neutralized with ethanol.

Contamination was a major limiting factor in the deposition setup. Examples of difficulties due to contamination include the loss of BuLi precursor when small amounts of air got into the reaction flask, and the repeating failure of the QCM measurement due to clogging and subsequent seal failure. Therefore, contamination was avoided as much as reasonably possible.

### Measures for safe handling of *n*-butyllithium

Due to the pyrophoric nature of *n*-butyllithium these safety procedures were followed. (i) The *n*-butyllithium solution was handled in an argon-filled glovebox, set up specifically for precursor handling. (ii) The precursor was prepared in the glovebox and transferred into a Schlenk flask that was resealed, thus ensuring inert atmosphere during transfer. (iii) A concentration of 10 mM of *n*-butyllithium in diethyl ether was selected such that the energy released during the exothermic reaction between n-butyllithium and atmosphere would be insufficient to ignite the diethyl ether. This approach was tested and confirmed to work. Notably, the last step of this approach increases the sensitivity of the precursor to contamination, which was a major limiting factor, as mentioned before. However, safety was chosen as a priority, and contamination can be worked around. We expect such an approach to be scalable for use with larger deposition chambers, using more sophisticated and contamination-proof precursor flasks and delivery.

### Deposition recipe

An example for a simple recipe used to deposit LiH: (i) The chamber was purged with pure solvent for 30 s. (ii) The solvent containing BuLi was pumped into the chamber for 10 s. (iii) The chamber was purged with pure solvent for 30 s. (iv) The solvent containing water was pumped into the chamber for 10 s.

Before, the deposition chamber was flushed with nitrogen for 1 min, and then with pure solvent for 2 min. The steps listed above were repeated for the desired number of cycles. After the last cycle was finished, a 1 min purge was performed to clean the chamber of any possible leftover precursors. Then the chamber was flushed with nitrogen from the Schlenk line, and only afterwards was the chamber opened. Since the deposition took place on the entire chamber, the film deposited on the glass slide could be used for further analysis. The chamber had to be cleaned with nitric acid after every deposition. Substrates of Si with native oxide and Pt/Si were used.

### Characterization

Auger spectroscopy was carried out on a Auger microprobe Jeol JAMP-9510F with hemispherical analyser using 3 kV accelerating voltage and 10 nA probe current. Sample was tilted 55° to the excitation, with the normal coincident with the axis of the collection optics of the analyser. Point Auger spectra were collected from different areas on the surface of the sample after 20 s cleaning with 500 eV Ar^+^ ions. Dwell time during the acquisitions was 100 ms with 1 eV measurement steps with an energy resolution of Δ*E*/*E* of 0.5 %. XPS signals were recorded using a Thermo Scientific K-Alpha XPS system (Thermo Fisher Scientific, UK) equipped with a micro-focused, monochromatic Al Kα X-ray source (1486.7 eV). An X-ray beam of 400 mm size was used at 6 mA and 12 kV. The spectra were acquired in the constant analyser energy mode with pass energy of 200 eV for the survey. Narrow regions were collected using the pass energy of 50 eV. Charge compensation was achieved with the system dual beam flood gun. The Thermo Scientific Avantage software, version 5.9904 (Thermo Fisher Scientific), was used for digital acquisition and data processing. Spectral calibration was determined by using the automated calibration routine and the internal Au, Ag and Cu standards supplied with the K-Alpha system. Argon etching was done with ion gun (1.4 µA of 2 keV Ar^+^ ions over 8 mm^2^).

The samples indented to be used in XPS and Auger were coated with an additional layer of SiO_2_ inside of the deposition chamber. This protective layer of about 2 nm was sputtered away during measurements. However, the protection was unsuccessful. O_2_ and CO_2_ diffused in through to the film, which was proven by XPS measurements showing Li_2_O and Li_2_CO_3_ after etching. This is described below in Table 1 and discussed further in subsection “Chemical Identity ”.

The surface compositions (in atom %) were determined by considering the integrated peak areas of the detected elements and the respective sensitivity factors. Grazing incidence X-ray diffraction was performed on a BRUKER D8 DISCOVER using the Cu Kα, at angle of incidence of 1°. The scan speed was changed as necessary. Ellipsometry measurements were performed using SENTECH SENpro ellipsometer, using the included halogen lamp. Frequency measurements on a quartz crystal were performed in situ using an OpenQCM module at 10 MHz.

## Results and Discussion

### Structure and possible reaction mechanism

Immediately after deposition, the chamber was opened, and the thin films visibly reacted with air, becoming whiter. This was markedly visible on the glass slides covering the chamber (Figure 2). During deposition, and before opening the chamber, the white film is not visible (Figure 3).

**Figure 4:**
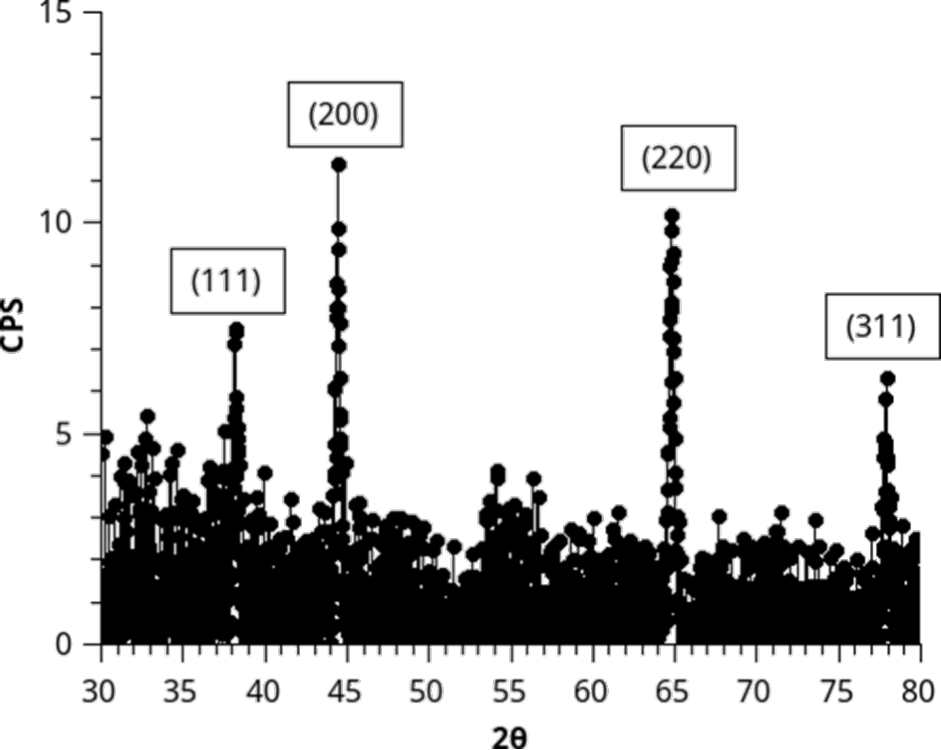
GIXRD of as-deposited samples with indexed reflections attributed to cubic LiH. GIXRD was performed at an incidence angle of 1°, and a scan speed 0.02°per 14.5 seconds.

Therefore, GIXRD was performed immediately after deposition. The GIXRD pattern of an as-deposited sample from the BuLi + H_2_O process reveals clear peaks of LiH as shown in Figure 4. Upon annealing at 600 °C peaks of Li_2_O appear as expected based on the reaction of LiH with oxygen from air.

It cannot be ascertained that the composition of the as-deposited films is pure LiH, since the reaction of H_2_O and BuLi classically produces LiOH, at least in the presence of water in excess. However, thermodynamic considerations demonstrate that in fact lithium hydride and the concomitant reaction byproduct butanol are more stable than lithium hydroxide (and butane). The Gibbs free energy of formation [14] of the possible reaction products is:


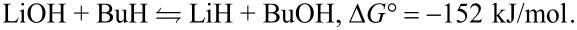


In other words, the driving force for generating LiH from BuLi and H_2_O is significantly larger than that for generating LiOH (unless a large excess of water is present to cause the subsequent reaction of the initially formed LiH under generation of H_2_).

**Figure 5:**
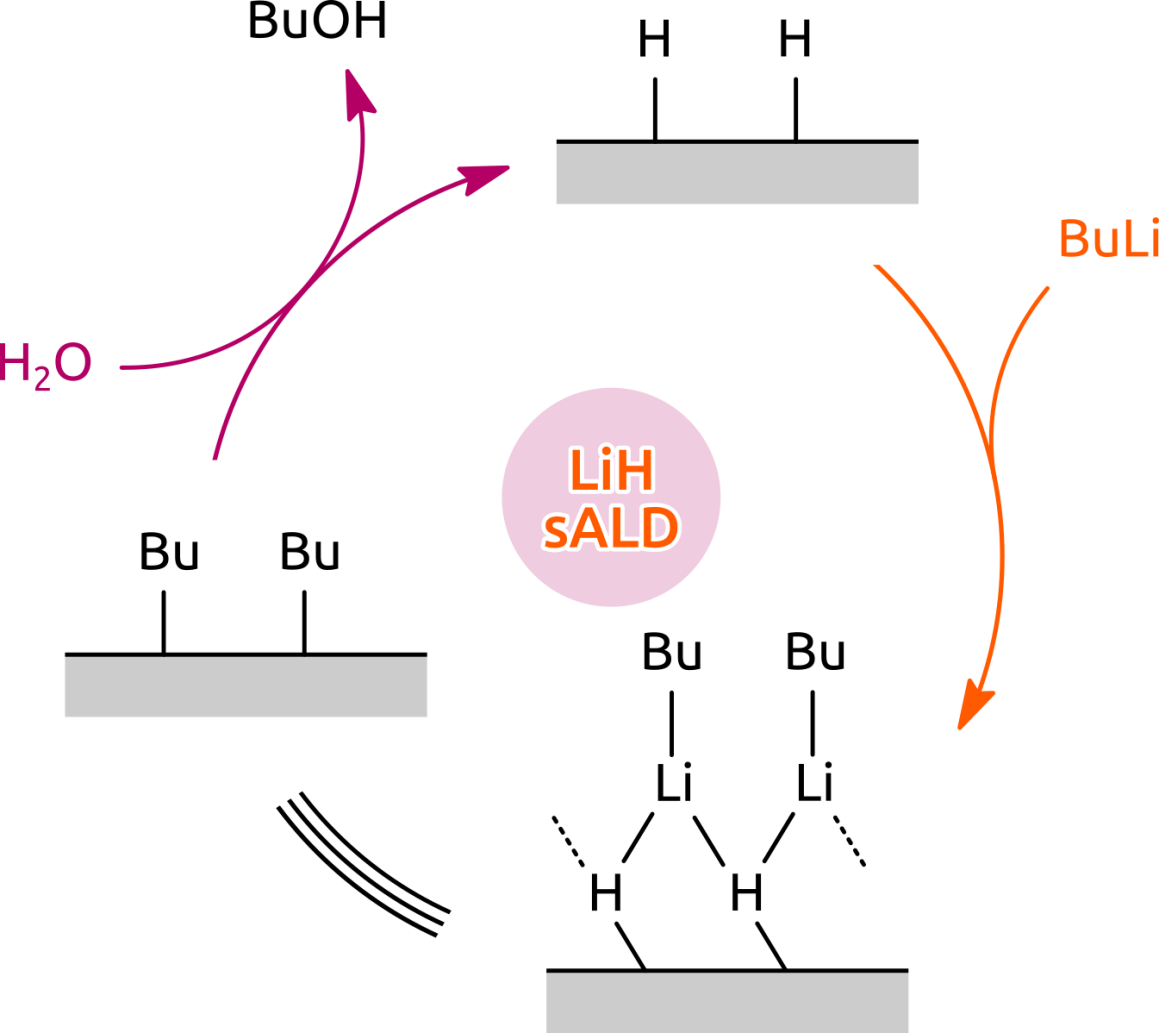
Possible mechanism of the surface reactions.

A likely reaction mechanism is sketched in Figure 5. The chemical identity of the surface alternates between hydride-terminated and butyl-terminated. While the water step releases butanol as a byproduct, the BuLi step results in a non-dissociative chemisorption of the precursor onto the surface.

### Chemical identity

The presence of LiH revealed through XRD needs to be confirmed by chemical analysis methods. Unambiguous analyses are rendered impossible by the air-sensitive nature of the deposit and the difficulty to identify the elements Li and H with techniques based on X-rays. In spite of this, the chemical nature of the film can be worked out from the presence of degradation products of LiH when combined with the XRD structural data. The exposure of LiH to ambient air generates two main degradation products [15–16] according to the reactions LiH + H_2_O → Li_2_O + 2H_2_ and 2LiH + CO_2_ + 0.5O_2_ → Li_2_CO_3_ + H_2_O.

**Figure 6:**
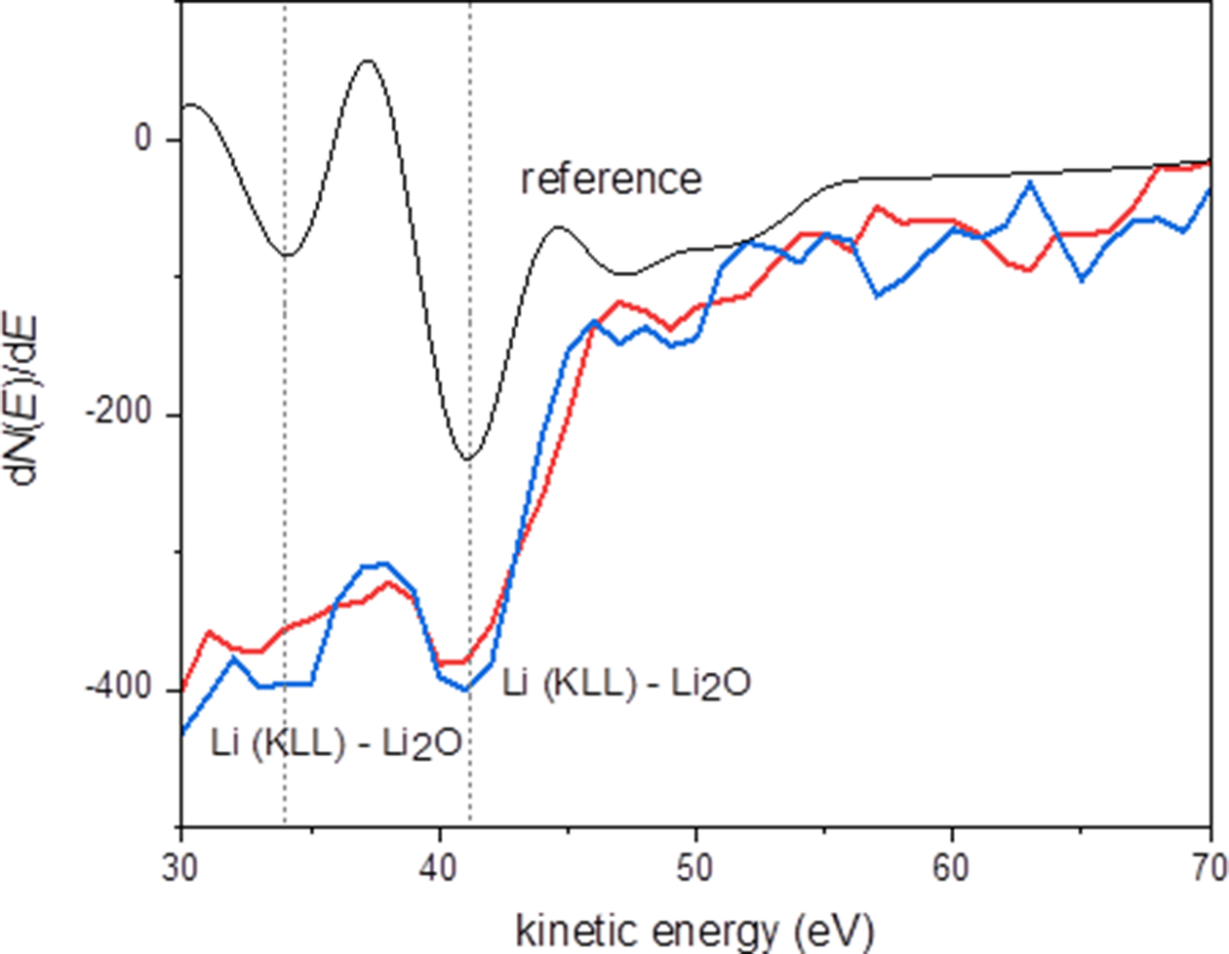
Derivative Auger spectra confirming the presence of Li_2_O on the surface, acquired at two different areas of the sample (red and blue), shown together with the internal reference for Li_2_O (black).

**Figure 7:**
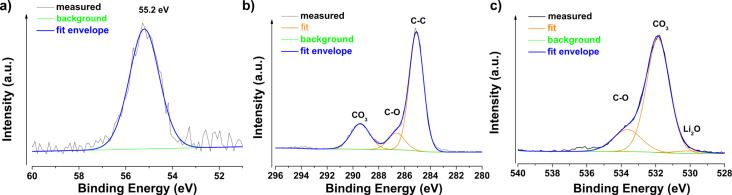
XPS spectra before Ar sputtering of a) Li 1s region, b) C 1s and c) O 1s region.

As expected, no LiH was measured on the surface by using Auger spectroscopy. However, the presence of Li_2_O is shown in Figure 6. Because Auger spectroscopy is technically able to detect LiH [16], we assume that the LiH was degraded completely by the time of the measurement. XPS, which cannot detect LiH, was carried out complementary to Auger spectroscopy, to measure the presence of Li_2_O and Li_2_CO_3_. XPS confirmed the presence of lithium on the surface and a Li 1s peak centred at 55.2 eV was detected (Figure 7a). This position of the Li 1s peak might correspond to Li_2_CO_3_[17] as well as to Li_2_O [18]. The C 1s spectrum exhibits three peaks shown in Figure 7b. The peak centred at 285.1 eV corresponds to C–C/adventiteous carbon, the second peak centred at 286.6 eV corresponds to C–O, and the third peak centred at 289.4 eV corresponds to the CO_3_ group [19]. These results are in agreement with the signals from the O 1s spectrum , shown in Figure 7c, with one peak at 531.9 eV (CO_3_ group) and one signal at 533.6 eV (C–O). Additionally, there is also a third small signal at 530.1 eV, which might correspond to Li_2_O [20]. This peak becomes more pronounced after the removal of the top 2 nm of the sample surface through Ar etching during XPS measurements (Table 1). After etching, also the stoichiometry of CO_3_ becomes more clear, because signals from surface contaminations overlapping this signal in the C 1s spectrum, such as carboxyl groups, were removed.

**Table 1:** Chemical composition of the sample surface as determined by XPS.

sample	surface chemical composition (atom %)		
	C 1s C–C/C–O/CO_3_	O 1s Li_2_O/CO_3_/C–O	Li 1s

surface	37.3/6.2/10.8	0.6/22.3/5.3	17.4
after etching (approx. 2 nm)	19.2/2.8/9.5	4.3/31.5/1.6	31.0

Table 1 shows that Li occurs mostly as Li_2_CO_3_. When subtracting the signals of Li_2_O (ca 2 atom % Li in Li_2_O, calculated from the O 1s signal) and Li_2_CO_3_ (21 atom % Li in Li_2_CO_3_, calculated from O 1s signal) from the total Li content calculated from the Li 1s signal, there is a difference of ca. 8 atom %. Hence, there might be LiH present in the layer. While contamination with oxygen is possible during deposition, by leaks or improper drying of solutions, a contamination from carbon during deposition was only possible if small amounts of CO_2_ leaked into the solvent vessels or the reaction chamber. The solvent itself acts as carrier and does not decompose. Decomposition products from the reaction between BuLi and diethyl ether could contaminate the films. This was avoided by always mixing fresh solutions before deposition. Therefore the carbon in the layer is assumed to come from the exposure of LiH films to CO_2_ in the atmosphere. Together, Auger and XPS measurements showed that the LiH film degraded into a mixture of Li_2_O and Li_2_CO_3_. Thus, when combined with the structural data obtained immediately after deposition, we can ascertain with high degree of confidence that the original film was indeed LiH.

### Growth behaviour

**Figure 8:**
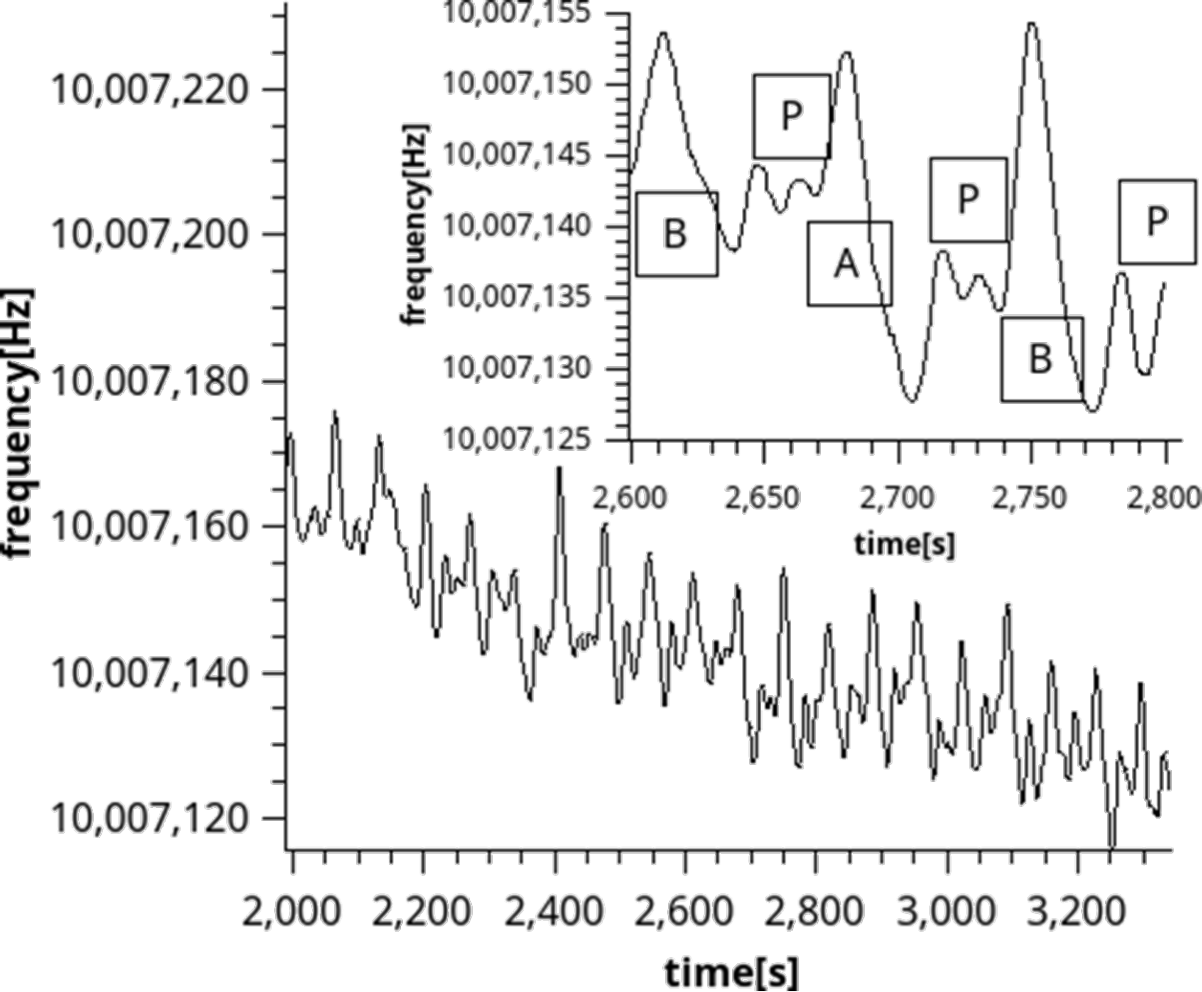
In situ QCM results, showing approximately 18 sALD cycles. The maxima and minima correspond to the timing of the sALD process. The inset shows the formation of small plateaus before the next peak. “A” corresponds to the BuLi flow, “B” corresponds to the water flow, and “P” corresponds to the purging.

QCM used during the deposition showed a linearly decreasing trend in frequency, clearly distinguishable from background noise (Figure 8). Moreover, the periodical changes also correspond to the changing cycles. The sharp increase in frequency during water cycles, marked “B” in the inset of Figure 8, is assumed to correspond to the relatively heavy butanol leaving the surface (see Figure 5). The plateau normally expected while using QCM to measure ALD reactions does not show clearly, possibly due to the connection of the QCM to the deposition chamber which causes a lag in the flow. However, a reasonable formation of plateaus, marked as “P” is shown in the inset of Figure 8. While longer purge times would lead to more pronounced plateaus, this would also mean an increased risk of contamination. In addition, the QCM was susceptible to leaks and blowbacks, therefore capturing a longer cycle proved to be difficult. Despite contamination being an issue preventing the use of QCM for saturation measurements, valuable information was gained from shorter cycle runs. The QCM results were not converted from frequency to mass, due to the complexity of the QCM crystal resonating in a fluid. The standard approximations for gases do not apply, and since both the surface and the fluid repeatedly changed, a more complex simulation would be necessary to obtain all the parameters necessary for converting frequency to mass [21–22]. Therefore the frequency change is shown, indicating and increase in mass with decrease of frequency [21].

**Figure 9:**
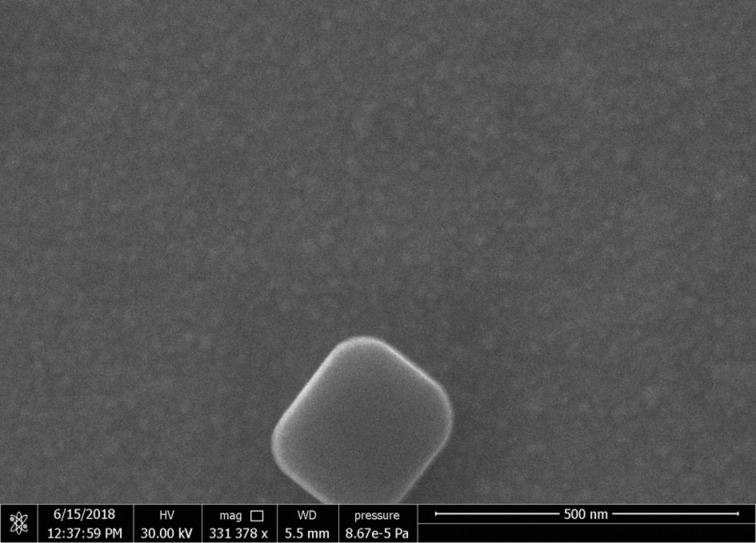
SEM of the Si/Pt sample surface after deposition and exposure to atmosphere. The large crystal seen is attributed to CVD growth in bubbles. The rough film is visible around the crystal.

SEM images of the surface show clusters of crystals that grew possibly due to bubbles forming in the chamber shown in Figure 3 or the influence of air sensitivity. One such crystal is presented in Figure 9. The film covering the sample appears to be homogeneous and rough as seen in Figure 9.

**Figure 10:**
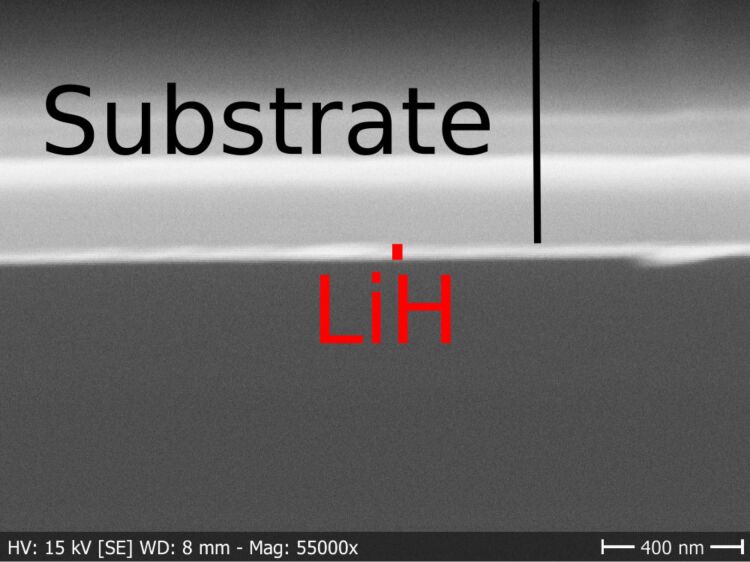
SEM of a Si sample cleaved after deposition. The film thickness was estimated to be roughly 45 nm.

**Figure 11:**
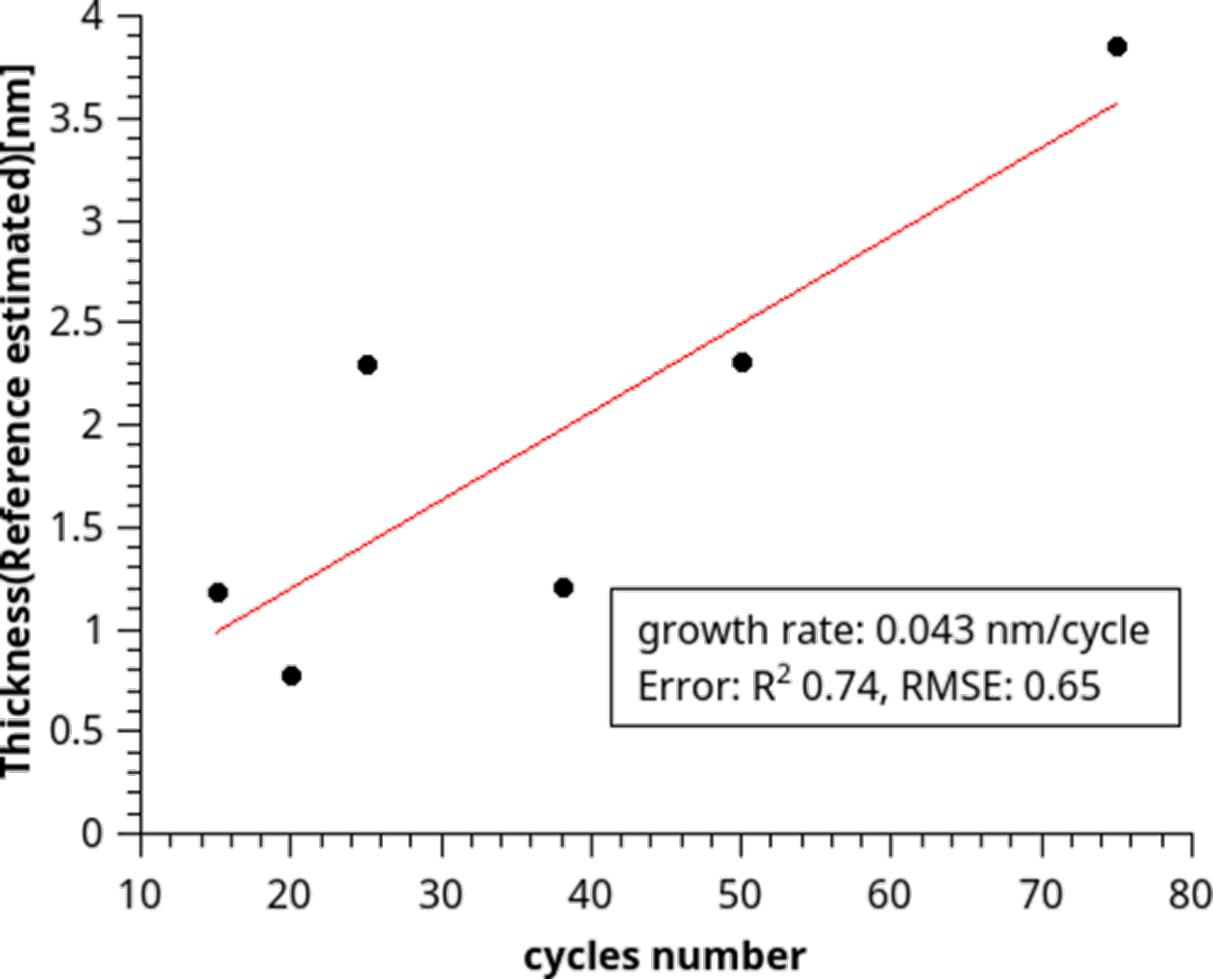
Growth curve obtained from spectroscopic ellipsometry. The thickness is relative to the SEM cross-section sample. The differences in thickness are correct.

Cross-sectional SEM was performed to estimate the film thickness as a reference for spectroscopic ellipsometry (Figure 10). Based on the SEM image, a Cauchy model was created with a constant thickness of 45 nm while all other parameters were fitted. This model was then used further, to enable ellipsometry measurements shortly after deposition. In this case, only the relative difference in thickness between samples could be measured, because the estimate from the cross-sectional SEM image was a rough estimate. Due to the relatively slow speed of the deposition and the high risk of contamination during long runs, only a few thick samples were prepared as cross sections. Spectroscopic ellipsometry measurements to determine the growth curve were performed directly after deposition (Figure 11).

The deviations from the fit, RMSE = 0.65, in Figure 11, are significant due to the instability of LiH films in air. For saturation this deviation causes the measurement to be indeterminate. In order to measure the saturation curve, further improvements of the sALD system are necessary, especially a construction of a glovebox-compatible sALD system. However, the growth rate (growth per cycle) of 0.43 Å/cycle is in line with expectations. Overall, the results indicate that an ALD process occurred with a definite growth per cycle, albeit its saturation has yet to be determined.

### Discussion

The experiment indicates that the reaction between BuLi and water can indeed create LiH, in agreement with theoretical considerations concerning the formation of LiH under dry, stoichiometric conditions. While a direct confirmation, through Auger spectroscopy measurements, of LiH would be desirable, the combination of structural data with the chemical identity of the degraded films has allowed us to be confident in claiming the film upon deposition was indeed LiH. Moreover, not only was it shown that the reaction produces LiH when used in the sALD deposition process, there are indications that the process is linear with respect to cycle number, according to the QCM measurements where the trend is a linear decrease in frequency. Ellipsometry measurements support the linearity of growth with cycle number as well, despite the larger error caused by the air sensitivity of the films. In addition, the films are crystalline as deposited, despite the fact that the deposition occurs at room temperature.

## Conclusion

To conclude, we have, for the first time, deposited LiH thin films by the sequential flooding of a deposition chamber with precursor solutions, more precisely, by using BuLi as a simple and highly reactive precursor. Furthermore, the films were crystalline when deposited at room temperature, making further post-processing unnecessary.

The air sensitivity of this solid requires in situ materials characterization using methods such as spectroscopic ellipsometry, XPS and Auger spectroscopy. Further development of the process, especially more sophisticated chambers that would allow one to work in a glovebox are necessary to further characterize the LiH deposition process. In particular, the measurement of the saturation curve without contamination is necessary to determine the nature of the process. The development of such new sALD setup would open up the possibility to research new growth processes that are also difficult to perform because of air-sensitivity.

Despite the yet undetermined nature of the process, this novel deposition method of LiH opens up the possibilities for further studies of using sALD in battery applications. Furthermore, LiH is not only suitable as an electrode material, where in fact it boasts the highest lithium concentration after elemental Li, it is also extremely useful as a hydrogen-torage layer. Since sputtering of LiH is possible [23–24], but not conformal, the sALD growth of LiH enables research regarding applications in electrodes, hydrogen storage [25], fuel cells [26–27], and neutron shielding [28].
